# TUSC3: a novel tumour suppressor gene and its functional implications

**DOI:** 10.1111/jcmm.13128

**Published:** 2017-03-08

**Authors:** Xinshuang Yu, Chunjuan Zhai, Yujun Fan, Jiandong Zhang, Ning Liang, Fengjun Liu, Lili Cao, Jia Wang, Juan Du

**Affiliations:** ^1^ Department of Radiation Oncology Shandong Provincial Qianfoshan Hospital Shandong University Jinan China; ^2^ Department of Cardiology Shandong Provincial Hospital affiliated to Shandong University Shandong University Jinan China; ^3^ Medical Management Service Center of Shandong Provincial Health and Family Planning Commission Jinan China; ^4^ Medical Research Center Shandong Provincial Qianfoshan Hospital Shandong University Jinan China; ^5^ China Institute of Veterinary Drugs Control Beijing China

**Keywords:** tumour suppressor candidate 3, oligosaccharyl transferase, tumourgenesis, *N*‐glycosylation reaction

## Abstract

The tumour suppressor candidate 3 (TUSC3) gene is located on chromosome region 8p22 and encodes the 34 kD TUSC3 protein, which is a subunit of the oligosaccharyl transferase responsible for the *N*‐glycosylation of nascent proteins. Known to be related to autosomal recessive mental retardation for several years, TUSC3 has only recently been identified as a potential tumour suppressor gene. Based on the structure and function of TUSC3, specific mechanisms in various diseases have been investigated. Several studies have demonstrated that TUSC3 is an Mg^2+^‐transporter involved in magnesium transport and homeostasis, which is important for learning and memory, embryonic development and testis maturation. Moreover, dysfunction or deletion of TUSC3 exerts its oncological effects as a modulator by inhibiting glycosylation efficiency and consequently inducing endoplasmic reticulum stress and malignant cell transformation. In this study, we summarize the advances in the studies of TUSC3 and comment on the potential roles of TUSC3 in diagnosis and treatment of TUSC3‐related diseases, especially cancer.

## Introduction

Tumour suppressor candidate 3 (TUSC3) was first identified on human chromosomes 8p22 by MacGrogan in 1996 1. It was found that the gene is highly expressed in a variety of epithelial cells and tissues including prostate, colon, lung, liver 1, ovary, placenta, testis and adipose tissues 2, but is rarely expressed in other tissues. Upon closer examination, more and more functions of TUSC3 have been discovered. TUSC3 interacts with the protein phosphatase 1 (PPPC1A) and affects the magnesium ion (Mg^2+^) transport system, which plays an important role in learning and memory 3. Therefore, the deletion or mutation of TUSC3 may be associated with non‐syndromic autosomal recessive mental retardation (ARMR) 4, 5. Required for cellular magnesium uptake, TUSC3 has been proven to be a pivotal molecule in embryonic development 2. Notably, TUSC3 has been identified as a potential tumour suppressor gene and is closely related to the malignant transformation of cells 3, 6. TUSC3 shares high sequence homology with Ost3p, a subunit of the oligosaccharyl transferase (OST) complex and is involved in the *N*‐glycosylation reaction of the protein folding process 7, 8, 9. The deletion or mutation of TUSC3 in cells may induce the accumulation of unfolding proteins and in turn the cells may suffer carcinogenesis 10, 11. In this review, we focus on recent results related to TUSC3's role in varied diseases and discuss the potential applications of TUSC3 in ‘TUSC3‐related diseases’, especially in cancers.

## The structure of the TUSC3 gene and protein

TUSC3 (GenBank accession No. AI366810), also named as N33, M33, MRT7, MRT22, OST3A and D8S1992, is located on chromosome 8p22 12. Chromosome 8 is a typical chromosome with respect to size (146.364 Mb), number of genes (1198), repeat content and degree of segmental duplication 13, 14. However, its p arm is a region with high frequent allelic loss and homozygous deletion 14. Recently, this region has been called an ‘anchor port’ contributing to cancer development through genomic alteration. It isthought to be a chromosomal region of frequent genetic loss 15.

**Figure 1:**
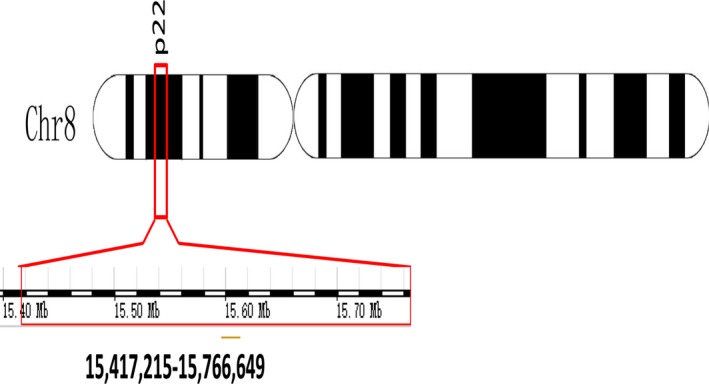
The TUSC3 gene locus.

**Figure 2:**
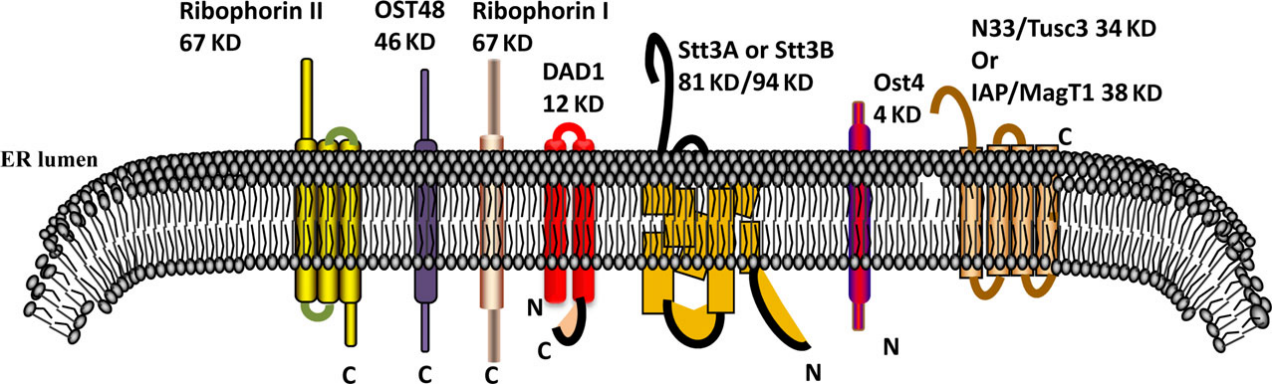
The structure of the oligosaccharyl transferase (OST) complex. ER Lumen, endoplasmic reticulum lumen.

TUSC3 gene is comprised of 11 exons spanning 349,435 bp of the genomic DNA on chromosome 8p22 (Fig. 1) 3. Two transcript variants encoding distinct isoforms have been identified for this gene (provided by RefSeq, Jul 2008). According to the UniProtKB database, TUSC3 encodes a predicted protein with 348 amino acid protein, including an N‐terminal region with 170 residues and four trans‐membrane regions. The TUSC3 protein is localized in the endoplasmic reticulum and is a subunit of the endoplasmic reticulum‐bound OST complex (Fig. 2), which is primarily responsible for protein N‐linked glycosylation catalysing the transfer of a 14‐sugar oligosaccharide from dolichol to nascent proteins during the process of protein maturity 4, 7, 8, 16. The human OST complex is a hetero‐oligomer of seven subunits 8 and consists of the subunits ribophorin I, ribophorin II, OST48, Ost4, DAD1, Stt3A or Stt3B and TUSC3/N33orIAP/MagT1 (Fig. 2) 16, 17. TUSC3 is believed to be the ortholog of the yeast Ost3p which is initially identified as a 34 kD subunit in the yeast oligosaccharyltransferase (OST) complex 18, 19. Two proteins share about 20% sequence identity, and both contain an N‐terminal active binding domain, central acidic and zinc‐binding domains, and a C‐terminal trans‐membrane domain 8. Its trans‐membrane segments and an N‐terminal signal peptide are particularly well conserved 16.

TUSC3 has two splicing variants that differ at their C‐terminal only in the last five residues 2. TUSC3‐1 co‐sedimented with the active OST complex, but TUSC3‐2 dissociated from the OST complex during membrane solubilization, and is most likely weakly associated with the active complex 20.

The subunits TUSC3 anchors to the ER membrane *via* C‐terminal trans‐membrane domains possesses its N‐terminal domains in the endoplasmic reticulum (ER) lumen, which includes a characteristic Cys‐Xaa‐Xaa‐Cys motif harbouring the active‐site cysteine pair 8. In normal cells, TUSC3 proteins have the capability of forming oligomers. It can delay oxidative substrate folding through mixed disulfide formation and increase the probability of sequon recognition and glycosylation by the catalytic subunit Stt3A/B as a nascent polypeptide chain passes the OST complex during co‐translational translocation 8. The unpaired cysteine residues of human glycoproteins have the potential to regulate glycosylation efficiency through transient interaction with TUSC3.

The similarities in primary and secondary structure between TUSC3 and yeast Ost3p suggest the existence of OST regulatory subunits in vertebrate cells 1. TUSC3 action in the regulation of *N*‐glycosylation is linked with its sequence‐specific interaction with polypeptide segments of glycoproteins. The glycan transfer process is a highly conserved process in eukaryocyte 17. Glycosylation is an important post‐translational modification in eukaryotic cells and has a significant impact on numerous biological processes 21. As N‐linked glycosylation of proteins is an indispensable modification for the correct folding of glycoprotein and onward transportation of newly synthesized proteins, the dysfunction or deletion of TUSC3 exerts its pathogenic effect as a modulator of glycosylation reaction in a variety of diseases, especially in tumours 17 .

## The relationship between TUSC3 and diseases

### Role of TUSC3 in maintaining normal function of the central nervous system

Many genomic imbalances on the 8p locus are associated with learning disabilities and have been touted as a ‘hub’ for neuropsychiatric developmental disorders 22, 23. The localization and structure of TUSC3 (located on 8p22) implies that it might be involved in the development and differentiation of the nervous system.

Early in 2008, Garshasbi 4 investigated seven patients with non‐syndromic ARMR in four sibling relationships. They found that the homozygous deletion caused a complete loss of TUSC3 function and was responsible for the observed non‐syndromic mental retardation (MR) phenotype. Then in 2011, Garshasbi 5 also found that independent mutations of TUSC3 cause a non‐specific form of severe ARMR. Khan 13 reported a novel mutation involving the deletion of the entire TUSC3 gene (except for the promoter and first exon) in a consanguineous Pakistani family with autosomal recessive non‐syndromic intellectual disability.

The above results suggest that TUSC3 plays an important role in maintaining normal function in the central nervous system. TUSC3 has proven to express ubiquitously in the foetal brain 7 and interact with the alpha isoform of the protein phosphatase 1 (PPPC1A) catalytic subunit, which is involved in the modulation of synaptic plasticity and in the memory and learning processes in mice 4. Other studies 2, 24 reveal that TUSC3 is also an Mg^2+^‐transporter involved in magnesium transport and is involved in magnesium homeostasis. Intracellular magnesium is abundant and plays an important role in biochemical functions and morphological and cytological changes 24. In fact, it has been demonstrated that TUSC3 is an indispensable member of the vertebrate plasma membrane magnesium ion (Mg^2+^) transport system 2 and the latest research results show that increasing Mg^2+^ level in the brain leads to an enhancement of learning abilities, working memory, and short‐and long‐term memory in rats 25.

**Figure 3:**
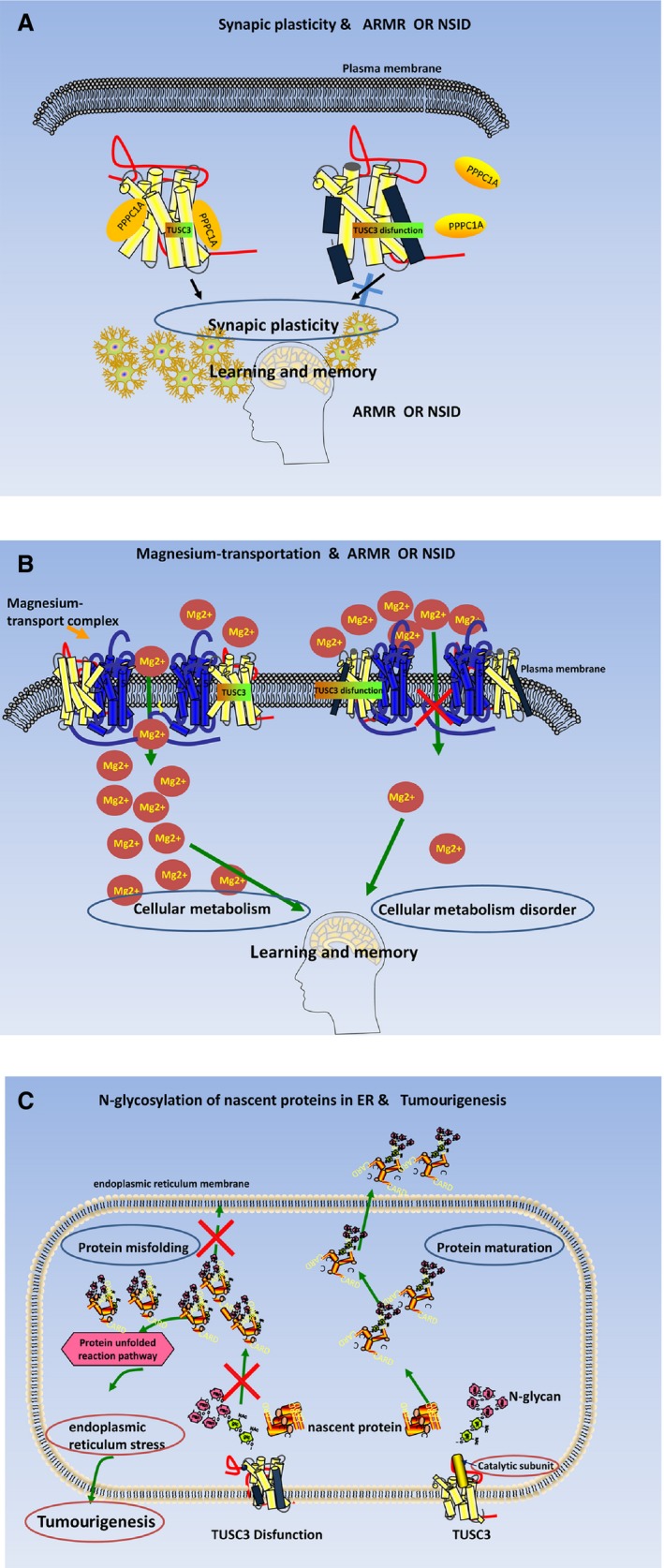
TUSC3 gene dysfunction in related to different diseases. (**A**) TUSC3 gene dysfunction in related to ARMR or NSID. Dysfunctional TUSC3 cannot interact with the alpha isoform of the protein phosphatase 1 (PPPC1A) catalytic subunit. Thereafter, the synaptic plasticity of neuron is impaired and in fact the memory and learning processes will be damaged. Consequently, the ARMR or NSID would occur. (**B**) TUSC3 gene dysfunction in related to ARMR or NSID. TUSC3 is an indispensable member of the vertebrate plasma membrane magnesium ion (Mg^2+^) transport system. Decreasing Mg^2+^ level in the brain leads to an impairment of learning abilities, working memory, and short‐and long‐term memory. Consequently, the ARMR or NSID would occur. (**C**) TUSC3 gene dysfunction in association with *N*‐glycosylation of proteins & Tumourigenesis. TUSC3 is a subunit of oligosaccharyl transferase (OST) complex which is responsible for protein N‐linked glycosylation catalysing the transfer of a 14‐sugar oligosaccharide from dolichol to nascent proteins during the process of protein maturity. TUSC3 disfunction induced the improper glycosylation and perturbation in protein folding, which in turn may induce alterations in endoplasmic reticulum structure and function, termed ER stress, and consequently result in malignant cell transformation. (ARMR), nonsyndromic autosomal recessive mental retardation; NSID, nonsyndromic intellectual disability; ER, endoplasmic reticulum.

Together, TUSC3 interacts with the alpha isoform of the protein phosphatase1 26, 27 and works with the magnesium ion transport system, which plays an important role in learning and memory 3 (Fig. 3A and B). It could be assumed that disturbed Mg^2+^ levels attributed to TUSC3 impairment were responsible for the MR phenotype observed in patients 5. Deficiency of human N33/Tusc3 results in isolated cognitive defects 4, 7. Further research on the functions of TUSC3 will provide significant insights into neuropsychiatric developmental disorders. Therefore, TUSC3 may be used as a helpful biomarker in the diagnosis of ARMR and as a therapeutic target for clinical treatment of ARMR in the future.

### Role of TUSC3 in maintaining normal function in embryonic development

Hao Zhou and David E. Clapham found that TUSC3 is engaged in embryonic development *via* interfering with cellular Mg^2+^ uptake 2. Studies 2 have proven that TUSC3 is indispensable to members with mammalian cellular Mg^2+^ uptake and is crucial for embryonic development in zebrafish. Reduction or deletion of TUSC3 expression in zebrafish embryos results in the early developmental arrest.

TUSC3 is also involved in spermatogenesis in the testis and is associated with sperm differentiation and maturation 24. Mg^2+^plays an important role in spermatogenesis, as magnesium administration can increases androgenic enzyme activity, which is crucial for spermatogenesis 28. As we know, sperm carries genetic cargo, which is necessary for systematic germ cell development and maturation. TUSC3 is upregulated during sexual maturation in the testis. Khalid *et al*. 24 demonstrated that TUSC3 was stronger in spermatocytes than spermatids. The TUSC3 gene participated in spermatogenesis *via* modulating the serum testosterone concentration, which is irradiated by modulating intracellular Mg^2+^ concentration.

However, the evidence that TUSC3 has influenced the embryo development and spermatogenesis is still inadequate. A deeper understanding of TUSC3's role in embryonic development and spermatogenesis requires further exploration.

### Role of TUSC3 as a tumour suppressor gene

Accumulated data have established a strong link between TUSC3 and cancer. It has already been proven that TUSC3 is either lost or reduced in several cancers, including prostate, ovarian, gastric, pancreatic cancer and osteosarcoma 1, 24, 29.

Early in 2005, Pils 30 reported that expression of TUSC3 was lower only in tumours of advanced grade (significant), FIGO (the International Federation of Obstetricians and Gynecologists) stages and in patients with relapse (trends not significant). This indicates that TUSC3 is correlated with cancer stage and invasive potential to some extent. Pils 6 proved that TUSC3 hypermethylation correlates with the survival of patients, indicating that TUSC3 is an accepted marker of poor prognosis in ovarian cancer patients. Moreover, another study 31 demonstrated that TUSC3 methylation level in blood leukocyte DNA was higher in gastric cancer patients than in the healthy controls, though with no statistic difference. Ribeiro 32 found that loss of TUSC3 gene may serve as a good indicator of malignancy in oral squamous cell carcinoma. TUSC3 gene played an important role in the transition from normal oral mucosa to potentially malignant oral mucosa. Loss of TUSC3 (8p22) is correlated with the advanced stage and lymph node metastasis and poor survival in larynx and pharynx squamous cell carcinoma 33. Our observations demonstrated that the TUSC3 expressions in normal controls were significantly higher than those in small‐cell lung cancer (SCLC) patients. Additionally, a marked decrease of TUSC3 expressions in patients with Lymph node metastasis positive (LNM^+^) was identified compared with patients with Lymph node metastasis negative (LNM^−^). Our results indicated that TUSC3 expressions may be a useful predictor of lymph node metastasis in SCLC cancer patients (Data not shown). The most significant cancer‐associated function of TUSC3 is associated with the vital role of OST catalysing in the protein *N*‐glycosylation process, which is tightly linked to the proper folding of nascent peptides shuttling and quality control 4, 16, 18.

*N*‐glycosylation of proteins 18 in the ER is a complex process and is crucial to ensure correct folding of newly synthesized proteins carried out by OST complex 4, 16, 34. It is well established that abnormal *N*‐glycosylation of proteins is associated with cancer development and progression 35, 36.

Perturbations in protein folding by improper glycosylation may induce alterations in endoplasmic reticulum structure and function, termed ER stress, and consequently result in malignant cell transformation (Fig. 3C) 17, 21.

Kratochvilova *et al*. 11 have reported that TUSC3 linked distinct biological mechanisms regulating the ER stress response and the EMT (Epithelial‐Mesenchymal Transition) *in vitro*, and promoting tumour growth *in vivo*. Vaňhara *et al*. 21 identified that loss of the TUSC3 promoted proliferation and migration of ovarian cancer cells through affecting the *N*‐glycosylating events in ovarian cancer. Peter Horak found that TUSC3 loss increased *N*‐glycosylation of cell surface proteins and alleviates endoplasmic reticulum stress in prostate cancer cells 10.

The precise molecular mechanism through which TUSC3 is involved in the development of cancer remains unclear. Some results demonstrate that TUSC3 is an ER integral protein involved in *N*‐glycosylation 21, 37. Previous studies have indicated that *N*‐glycosylation abnormalities affect the growth of tumour cells by affecting the PI3K‐Akt pathway 38. Perhaps there are other pathways remaining to be found.

## Mechanisms for TUSC3 gene dysfunction

Frequently deletion, mutation and promoter hypermethylation are common mechanisms for gene dysfunction 39. The dysfunction of TUSC3 was also commonly because of the genetic and epigenetics alterations 6.

### Genetic alteration of TUSC3

TUSC3 was first cloned from a homozygous deletion in a metastatic prostate carcinoma 40. Homozygous deletions of the TUSC3 gene have been revealed *in vitro* experiments on human prostate, lung, liver and colon cell lines, as well as lymph node tumour, breast and pancreatic cancer 24, 39, 41. Loddo 42 reported on a boy affected by ‘syndromic’ ID (Intellectual Disability) with a homozygous micro‐deletion in 8p22, encompassing the first exon of TUSC3 in which the alteration consists of an intragenic deletion. Ghadami 43 also found a high percentage of homozygous deletions of TUSC3 in the Iranian population. Homozygous loss and homozygous mutations of TUSC3 occur mostly in the family tree of close relatives 13. Aberrant TUSC3 reduction in many cancers was detected. The sites of the deletions mutation was identified within exon 1 region 14 and nonsense mutation was within exon 2 region 5.

Frequently, loss of heterozygosity of TUSC3 was found in several diseases. Sleptsov 44 demonstrated the heterozygosity loss of TUSC3 in vascular tissues and peripheral blood leukocytes from patients with atherosclerosis. TUSC3 has already been suggested as an important target of genomic rearrangements in epithelial cancers 45. Copy‐number variation involving TUSC3 has also been described in a parent and child from the Hap Map project 46, 47, 48, 49. Alternative splicing is an important component of tumourigenesis with indication of known alternative splicing in TUSC3 50. Additionally, the TUSC3 gene single nucleotide polymorphism was found in patients with non‐syndromic MR 3. Major mutations/deletions reported to date as pertaining to different diseases/cancer are illustrated in Table 1.

**Table 1:** The mutation patterns found in TUSC3 in diseases/cancers

Diseases	Mutation patterns	TUSC3 functions and clinical significance	References
NS‐ARMR	Homozygous deletion	A defect in the TUSC3 gene is associated with NS‐ARMR	4
NS‐ARMR	Nonsense mutation (c.163C>T [p.Q55X])	TUSC3 mutations cause an non‐specific form of severe mental retardation	5
NS‐ARMR	Missense mutation (c.932T/G, p.V311G)	TUSC3 mutation is associated with NS‐ARMR	7
NS‐ARMR	Homozygous deletion	A novel deletion mutation in the TUSC3 gene in a consanguineous Pakistani family with NS‐ARMR	13
NS‐ARMR	Homozygous aberrant transcript at Exon 7	Homozygous Truncating Intragenic Duplication in TUSC3 Responsible for NS‐ARMR	38
Prostate cancer	NM	Loss of TUSC3 expression in prostate cancer cell lines leads to increased proliferation, migration and invasion as well as accelerated xenograft growth.	10
Ovarian cancer cells	Loss of TUSC3 (NM)	Loss of TUSC3 enhances proliferation and migration of ovarian cancer cells *in vitro*.	21
Ovarian Carcinoma	Loss of TUSC3 (NM)	Expression of N33 has an impact on survival and tumour grade	31
Oral squamous cell carcinoma	Homozygous deletions	Loss of TUSC3 gene may serve as a good indicator of malignancy.	32
Larynx and pharynx carcinomas	NM	Loss of TUSC3 was correlated with positive lymph node as well as a worse impact on larynx–pharynx carcinoma survival	33
Pancreatic adenocarcinoma	Heterozygous deletion	NM	42
Primary breast tumour	Copy number loss within 12.7 Mb‐19.1 Mb in 8p22	NM	46
Non‐small cell lung cancer	Alternative splicing	NM	51

*NS*‐ARMR, *Nonsyndromic autosomal recessive* mental retardation; NM, no mention.

### Epigenetics alterations of TUSC3

Silencing of TUSC3 expression can also occur by CpG methylation within the promoter. This mode of TUSC3 expression silencing is a frequent occurrence in cancer cells. DNA methylation regulates gene expression by influencing the chromatin structure and the accessibility of DNA, which are among the most common molecular alterations in human cancers and other diseases 51, 52 .

Aberrant hypermethylation of CpG islands in tumour suppressor genes can result in their silencing in cancer, while hypomethylation can lead to increased oncogene expression 53, 54. Local hypermethylation (at the promoter CpG island) has been well established for the inactivation of tumour suppressor genes and has attracted attention in the scientific community 55.

TUSC3 is a defined tumour suppressor gene and methylation usually occurs in its CPG island promoters. Reduction of TUSC3 gene expression in prostate cancer may be because of its methylation 10, and some of these methylation changes may initiate in subpopulations of normal cells as a function of aeging and progressively increase during carcinogenesis 56. In colon cancer, it was found that TUSC3 reduced and showed a pattern of age‐related methylation 56. Another research by Pils 6 showed that TUSC3 expression decreased significantly because of promoter methylation in malignant ovarian tumours when compared with benign controls. Furthermore, TUSC3 promoter methylation showed an association with pre‐eclampsia 57. The TUSC3 methylation patterns are illustrated in Table 2.

**Table 2:** The methylation patterns found in the TUSC3 gene

Diseases	Methylation patterns (site)	Clinical significance	References
Prostate cancer cell lines, liver cancer cell lines, lung cancer cell lines, colorectal cancer cell lines	Exon 1 CpG islands	NM	1
Gastric cancer	NM	TUSC3 methylation level was higher in the cases than in the healthy controls, but the difference was not significant	31
Ulcerative colitis	The promoter CpG islands	TUSC3 methylation may not be associated with colorectal carcinogenesis in ulcerative colitis	53
Breast cancer	Promoter methylation	The methylation state of TUSC3 may be associated with worse survival in African American CBCS	55
Colorectal carcinogenesis	Promoter CpG islands	TUSC3 expression displayed no tumour associated changes	10
Ovarian cancer	Promoter methylation	TUSC3 Promoter Methylation Predicts Survival in Patients With Ovarian Cancer	57

In a word, further exploration of the causes of TUSC3 dysfunction, including genetics changes and epigenetic alterations, will promote an increased understanding of the causes of TUSC3‐dysfunction‐related diseases.

## Concluding remarks

Since its first identification in 1996, TUSC3 has become more and more involved in the researches of human diseases. Defects in the TUSC3 gene have been identified in individuals with non‐syndromic autosomal recessive intellectual disability in different nations. Recently, researchers recognized it as a tumour suppressor gene that is negatively related to the malignant transformation of cells. Owing to its functional importance in the process of protein maturity, the abnormal expression of TUSC3 may be the critical cause of relative diseases, such as ARMR, oral squamous cell carcinoma, ovarian cancer, *et al*. It is believed that TUSC3 may be used in the future as a helpful biomarker and as a therapeutic target in the diagnosis and therapy of ARMR and related cancers.

Regarding the various possibilities for the therapeutic targetting of the TUSC3 protein in diseases, researches on drug discovery to reinstate TUSC3 function in diseaseswill mainly focus on three avenues: (1)TUSC3 gene therapy, for example, recombinant Ad‐TUSC3 gene therapy, (2) targetting the TUSC3interactive proteins, inparticular negative regulators of TUSC3, and (3) targetting destabilized oncogenic TUSC3 mutants, that is to design mutant‐specific TUSC3 rescuedrugs. However, many structural aspects of TUSC3's function have remained elusive, such as the details of TUSC3's interactive proteins and the exact knowledge of TUSC3 mutations in diseases. TUSC3 targetting therapy has a long way to go.

## Conflict of interest

The authors declare that we have no conflict of interest.
